# Fast, label-free super-resolution live-cell imaging using rotating coherent scattering (ROCS) microscopy

**DOI:** 10.1038/srep30393

**Published:** 2016-07-28

**Authors:** Felix Jünger, Philipp v. Olshausen, Alexander Rohrbach

**Affiliations:** 1 Laboratory for Bio- and Nano-Photonics, Department of Microsystems Engineering, University of Freiburg, Germany; 2 Testo AG, Testo-Straße 1, 79853 Lenzkirch, Germany; 3 BIOSS Centre for Biological Signalling Studies, University of Freiburg, Freiburg, Germany

## Abstract

Living cells are highly dynamic systems with cellular structures being often below the optical resolution limit. Super-resolution microscopes, usually based on fluorescence cell labelling, are usually too slow to resolve small, dynamic structures. We present a label-free microscopy technique, which can generate thousands of super-resolved, high contrast images at a frame rate of 100 Hertz and without any post-processing. The technique is based on oblique sample illumination with coherent light, an approach believed to be not applicable in life sciences because of too many interference artefacts. However, by circulating an incident laser beam by 360° during one image acquisition, relevant image information is amplified. By combining total internal reflection illumination with dark-field detection, structures as small as 150 nm become separable through local destructive interferences. The technique images local changes in refractive index through scattered laser light and is applied to living mouse macrophages and helical bacteria revealing unexpected dynamic processes.

The smaller a structure, such as a cell organelle, a filament or a molecule, the faster it can move because of less friction. Motions inside a living cell, driven either by thermal energies or ATP hydrolysis, are not band-limited, i.e., exist on all time- and length-scales. For example, globular structures on a scale of a few ten nanometres move or change their shape on a microsecond timescale. Although super-resolving microscopy techniques have increased in speed and image contrast^1^,^2^,^3^,^4^,^5^ or have forged ahead to image structures of a sub-100 nm length scale^6^,^7^,^8^,^9^,^10^, they all have the problem that the image acquisition process needs much time or many photons or both. In other words, the requirements for investigating small dynamic structures and the requirements for super-resolution imaging are far from overlapping.

Most live cell imaging methods providing high and specific contrast are based on fluorescence and are therefore subject to bleaching. Especially for super-resolving techniques, the blackout of fluorophores limits long-term investigations, where often hundreds of images need to be acquired. Fluorescence microscopes offering super-resolution, high contrast, high acquisition speed, which are all maintained for a large number of acquisitions, do not yet exist.

When specific labelling of structures and molecules is not the main issue, coherent imaging techniques based on the scattering of the illumination light at cellular structures can meet all the above-mentioned shortcomings. However, imaging with coherent illumination light, e.g., from lasers, is known for pronounced interferences between the incident and the multiply scattered light and for the resulting strong image artefacts. Usually, any information about the cell’s structure is disfigured and can hardly be recovered from coherent images. Different to imaging, the interference intensities between unscattered light and light scattered at single particles can be easily analysed allowing to track their positions with nanometre precision at high temporal rates^11^,^12^,^13^. Coherent imaging was demonstrated only recently by using digital holographic microscopy in combination with extensive post-processing, enabling super-resolution imaging even of bacteria and cortical neurons^14^. In a similar approach with a rotating laser, but based on a different contrast formation and without post-processing^15^, we could demonstrate high-contrast super-resolution imaging of a 2D layer of small, fixed latex beads by rotating coherent scattering (ROCS) microscopy in combination with total internal reflection (TIR) and dark field (DF) illumination^16^.

In this article we show that despite multiple scattering of the laser illumination light it is possible to record super-resolved images of living, highly dynamic cells based on multiple interferences. The images of adherent J774 mouse macrophages and of swimming helical bacteria offer an exceptionally high contrast, while thousands of images are recorded at 63 and 100 Hz without any loss in image quality and without any post-processing.

## Results

The realization of our imaging technique is quite simple, it can be added to most inverted microscopes and can be combined with most other standard microscopy techniques.

### Experimental Setup and operation principle

**Figure 1:**
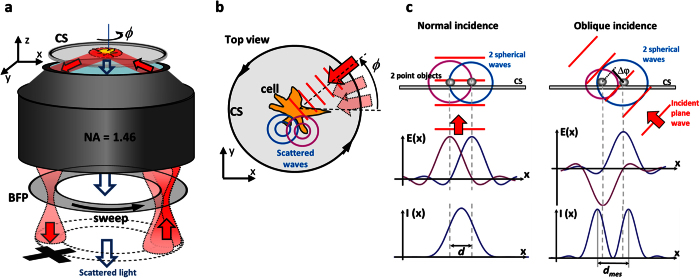
Experimental setup and operation principle. (**a**) A focused laser beam is scanned along a circle in the back focal plane (BFP) of the objective lens. The object on the coverslip (CS) is illuminated by an oblique plane wave, which is totally internal reflected (TIR-Mode). The scattered light is transferred to the camera, whereas the reflected beam is blocked to enable dark field imaging. (**a**,**b**) Within the integration time of the camera, the laser sweeps a *ϕ* = 0…2π angle. (**b**) Each plane wave, indicated by the red wavefronts, is multiply scattered at the object (the cell), indicated by two spherical waves (in blue and purple). For each angle *ϕ*, a (radially) coherent image with many artefacts is generated. A superposition of many different coherent images within the integration time of the camera generates a (tangentially) incoherent image with hardly any artefacts. (**c**) For normal incidence, two scatterers in a short distance *d* < *λ*/2 excite two spherical waves, whose electric fields E(*x*) are in phase at the camera. The two points cannot be resolved by the resulting intensity *I*(*x*) at the camera. For oblique incidence, the two scatterers excite spherical wavefronts with a phase delay Δ*φ* = π. Two maxima in a distance *d*_*mes*_ can be resolved in *I*(*x*).

Simply spoken, the ROCS technique just requires a focused laser beam, which circulates in the back focal plane of high *NA* objective lens. The angular laser sweep frequency has to be synchronized with the desired integration time of the camera, corresponding to a frame rate which can be several hundred Hertz if a fast camera is available. Since the ROCS technique has so far only been examined in 2D imaging, we decided to use TIR-illumination to have access to all object structures in a 2D plane, in combination with dark-field detection to remove the unscattered TIR light. Therefore, any standard TIR setup equipped with a lens with *NA* > 1.33 is suitable. To achieve coherent illumination, a laser beam needs to be focused into the outer part (the TIR ring) of the back focal plane (BFP) of the objective lens. As illustrated in Fig. 1a, this results in a collimated beam incident under a supercritical angle and thereby in a completely reflected beam (TIR). The evanescent field on the upper side of the coverslip (CS) interacts with the unlabelled sample and the downwards scattered light is collected with the camera. To enable dark-field illumination, the totally reflected beam is blocked in a plane conjugate to the BFP, which is realized by another 4*f* system and a diaphragm, as shown in Supplementary Figure S1 and indicated by the black cross in Fig. 1a. The circular scanning of the focused laser beam is achieved by a two axis tilt mirror (for details see Supplementary Text 1).

During the integration time of the camera, which is typically a few milliseconds, the laser circulates in the BFP and sweeps the full azimuthal 2π angle expressed by *ϕ*. In other words, during this time, the object is illuminated from all directions and light is multiply scattered from all directions, as pointed out by the red wave vector and plane wave fronts in Fig. 1b. The integration of the camera over one sweep period results in an averaged image, which strongly suppresses the unwanted scattering artefacts known from coherent imaging. The question arises, why coherent illumination is so beneficial?

### The coherent super-resolution principle

Already in 1873 Abbe realized^17^ that oblique illumination of an object increases the optical contrast and resolution through a shift of the frequency spectrum. This concept was extended by Cronin^15^ 100 years later by averaging the illumination from all azimuthal directions. This concept, which increases the contrast (the optical transfer) at high spatial frequencies is related to other super-resolving coherent imaging approaches published recently^14^,^16^,^18^,^19^,^20^. In incoherent imaging, object spectra (i.e., the spatial Fourier transform of the object function) are shifted by modulated object illumination^21^, a technique well-known as structured illumination microscopy (SIM)^22^,^23^. The principle of super-resolution by oblique illumination is based on a small phase delay between spherical waves emitted by two adjacent objects or parts of a structure, as indicated in Fig. 1b,c. When the two point objects are excited to emit waves at the same time (without phase delay) by an incident plane wave under normal incidence as shown in Fig. 1c, the response patterns of the electric fields **E**(*x*) interfere constructively in the camera plane. In the resulting interference intensity *I*(*x*), the two objects in a distance *d* cannot be resolved. However, for oblique incidence, the two objects are excited with a phase delay of Δ*φ* ≈ π (see Fig. 1c), such that the electric fields from the emitted spherical waves in the camera plane interfere destructively. In consequence, the two distinct intensity lobes in a distance *d*_*mes*_ make the objects resolvable. The measured distance in the image plane *d*_*mes*_ ≈ *d* + Δ*d*, is a few percent broader than the actual distance *d* and is an interference effect depending on the phase delay Δ*φ*. The non-global, i.e., local effect that two adjacent amplitude point spread functions are out of phase and overlap such that their sum intensity reveals two peaks in a slightly increased distance, is shown in the bottom right of Fig. 1c.

### Multiple interferences increase image quality

Super-resolution, the separation of two adjacent objects in a distance *d* < *λ*/2, can be achieved by inducing local destructive interferences between adjacent structures (scatterers). This requires coherent illumination, where amplitudes of the electromagnetic field add up to generate a coherent image. Now, a complex object, such as a biological cell, can be thought of to be composed of many small structures (scatterers) of different shapes and different refractive indices. The light scattered at all these structures can be measured at the camera, each with a different amplitude *A*_*j*_ and with a different phase delay *φ*_*j*_ depending on their position on the coverslip (see the two points in Fig. 1c right). The electric field image *F*_*j*_ of the *j*-th scatterer at position **r** illuminated from direction *ϕ* can be written as *F*_*j*_(**r**, *ϕ*) = *A*_*j*_(**r**, *ϕ*) exp(−*iφ*(**r**, *ϕ*)). The intensity distribution *I*(**r**) of the final TIR-ROCS image formed by multiple interferences resulting from the coherent illumination of a distribution of point scatterers can be expressed as follows^16^:


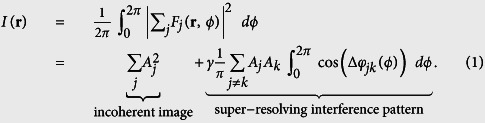


The first term in the second line represents the incoherent image of the object. The second term represents the multiple interferences and sums the phase differences Δ*φ*_*jk*_(*ϕ*) between pairwise point-scatterers over all illumination directions *ϕ* weighted with a cosine. This sum is generally not zero but depends crucially on the locations **r** of the scatterers *j* and *k*, on the direction of illumination *ϕ* and on the degree *γ* of temporal and spatial coherence of the illumination light.

Two adjacent scatterers *j* and *k* can be resolved at best when excited with a phase delay of Δ*φ*_*jk*_ ≈ π, 3π, etc. relative to each other. For Δ*φ*_*jk*_ ≈ 0 or Δ*φ*_*jk*_ ≈ 2π, 4π, etc. no contrast is generated on the camera. However, also a single small scatterer generates contrast since it delays the phase by Δ*φ* ≈ π/2 relative to the incident field^24^. In this way, our technique can also enhance the contrast at edges of the objects (e.g., cells), or more precisely at refractive index changes. A coherent image contains an interference pattern (2^nd^ term in equation (1)) in addition to the incoherent image (1^st^ term in equation (1)), which can be a conventional fluorescence image for instance. Therefore, the TIR-ROCS image is a superposition of the conventional image with a complex interference pattern, which provides the higher resolution and contrast.

### Image generation through circular scanning

Since no contrast is generated between the images of scatterers placed aside each other, but only of scatterers behind each other, one needs to illuminate the distribution of scatterers (the cell) from all directions, to ensure that two adjacent scatterers are behind each other from at least one direction. Therefore we scan the cells from all sides with incident angles *ϕ* = 0…2π.

**Figure 2:**
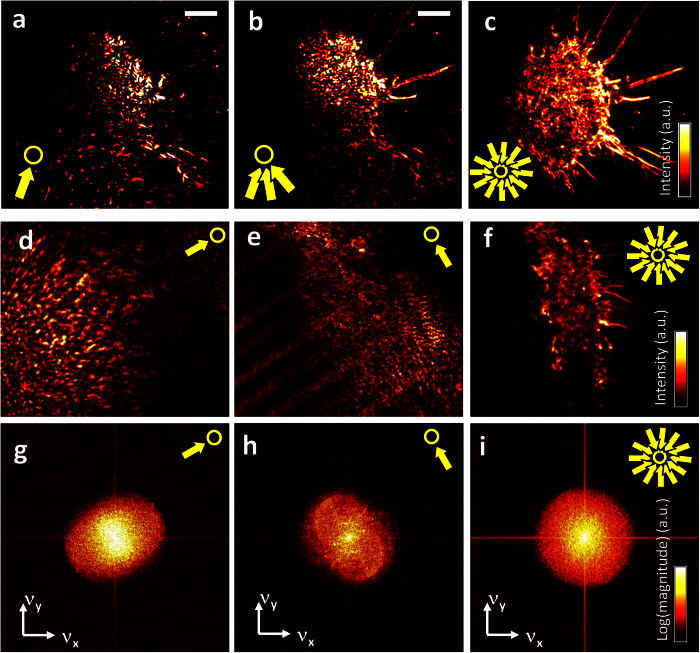
Generation of images and spectra in TIR-ROCS. (**a**,**b**) Stepwise superposition of images generated by illumination from defined angles. Yellow arrows indicate the direction of illumination, the number of arrows denote the number of images that were summed up. Artefacts resulting from coherent illumination become less apparent, cellular structures become better visible with each additional angle. (**c**) The final image of a living J774 macrophage results from incoherent addition of all intensity distributions generated from different illumination directions (0°…360°) within 16 ms. (**d**,**e**) Individual images from a single illumination direction hardly reveal any cell details, whereas the corresponding image spectra show a significant broadening above noise in this direction (**g**,**h**). Final image of cell (**f**) with broadened, round spectrum (**i**). Scale bars: 5 μm.

This principle is illustrated in the image sequence of Fig. 2a–c, where a living J774 mouse macrophage was illuminated in TIR-mode by a laser beam of 488 nm wavelength. The first figure shows the coherent image of the cell when illuminated from a single direction, indicated by the yellow arrow. The second image is generated by three illumination directions (see three arrows), where the individual images add up incoherently, the third image is illuminated from all directions *ϕ* = 0…2π. It can be seen that the amount of scattering artefacts (speckles) is reduced with increasing number of illumination directions and features of the cell, such as filopodia, become more prominent. Figure 2c displays the final image of a macrophage with adherent filopodia, which has been illuminated from all directions within the camera integration time of 16 ms. The image formation process is not affected by small time delays or by the absolute angular position *ϕ*_*0*_ at which the circular scan starts. Only must the camera acquisition time match the 2π scan period of the mirror.

For a sequence of final images as shown in Fig. 2c,f, a single background image without the cell is taken and subtracted from the images with the cell (see Supplementary Text 2 and Figure S2).

### Coherent image formation under oblique illumination

A TIR-ROCS image *I*(**r**) is obtained by averaging over many coherent images *I*(**r**, *ϕ*) from different directions *ϕ*, i.e. through integration over a 2π angular sweep: 
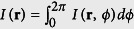
. For the image formation, the refractive index distribution *f*(**r**) of the object is illuminated with a laser beam incident from direction **k**_**i**_, with electric field 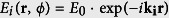

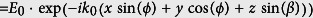
. Hence, the intensity for a single coherent image is obtained by convolution (*) with the coherent point spread function *PSF*_*coh*_(**r**):


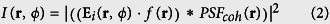


Two exemplary images are shown in Fig. 2d,e. The Fourier transform 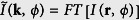 is obtained by the autocorrelation *AC* of the amplitude image spectrum, which is the product of the coherent optical transfer function 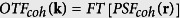 and the object spectrum shifted by the incident k-vector, *F*(**k**−k_*i*_) = *F*(**k**)*δ(**k**−**k**_*i*_) = *FT*[*f*(**r**) **E**_*i*_(**r**,ϕ)]. We find for a single image intensity spectrum:


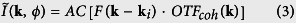


**Figure 3:**
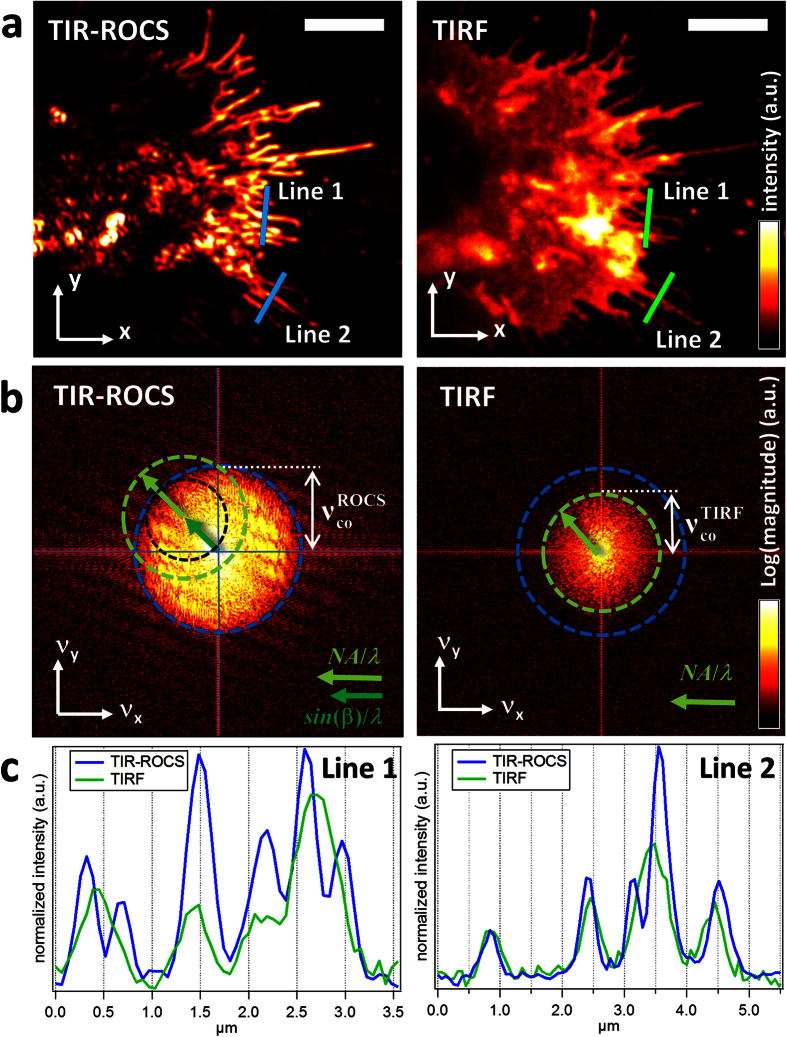
Comparison between TIR-ROCS and conventional TIRF. (**a**) J774 cell with adherent filopodia. Left: TIR-ROCS, right: TIRF mode image of the same sample. Scale bar: 5 μm. (**b**) The modulus of the Fourier transform of the images shown in (**a**) reveals the improved transfer of spatial frequencies, which are limited by the green circle (TIRF) and the blue circle (TIR-ROCS). The black circle is about 20% smaller than the green circle and accounts for the low pass filtering by the dark-field diaphragm. (**c**) Comparison of intensities along line profiles as indicated by the coloured lines in (**a**).

The coherent optical transfer function is nothing else but a segment of the (Ewald) spherical cap. Through multiplication, the *OTF* selects frequencies out of the object spectrum, which are the higher, the longer the additional vector **k**_**i**_. In this way object information becomes available, which is not accessible with perpendicular illumination (**k**_**i**_ = 0). This is illustrated in Figs 2 and 3 and in more detail in Supplementary Figure S3. Figure 2g,h show the enhanced optical transfer of the image spectrum as a consequence of the shift of the object spectrum *F*(**k**−**k**_*i*_) by **k**_**i**_ = 2π · **ν**_i_.

### Comparison to fluorescence imaging

The image quality was investigated in comparison to that of fluorescence images, which were acquired in TIRF-Mode with a *NA* = 1.46 lens. We used fixed J774 mouse macrophages labelled with a membrane dye (WGA-staining) to ensure that adherent filopodia can be optimally imaged (see Supplementary text 4). Images were acquired first in TIRF mode and then in TIR-ROCS mode. While the exposure time in TIR-ROCS mode was merely 20 ms, TIRF images were taken with 5 seconds integration time to achieve a comparable image contrast. Figure 3a displays both results side-by-side: on the left, the image of the cell generated purely by scattered laser light, on the right, the same cell imaged through fluorescence of the membrane. The overall shape of the cell appears similar with both imaging concepts, the distribution of filopodia is nearly identical. However, whereas in the TIRF image the whole cell membrane is visible as expected, the TIR-ROCS image presents a distribution of local refractive index changes, which corresponds to a reasonable approximation to the local changes in the density of scattering protein structures at the periphery of the cell. In the case of a filopodium, for instance, this is the actin bundle and the lipid membrane.

The contrast and resolution in both images *I*(*x*, *y*) is compared globally in Fig. 3b by the modulus of the Fourier transform *Ĩ*(*v*_*x*_, *v*_*y*_), and locally in Fig. 3c by intensity line scans at the cell periphery. Both Fourier transforms are normalized to the same maximum and minimum to allow a fair comparison. The magnitude of *Ĩ*(*v*_*x*_, *v*_*y*_) which is 10% above the mean noise level was used to define the cut-off frequency, which represents the maximal spatial frequency transferred by the imaging mode. The cut-off frequency, which is only a rough indicator for the optical resolution of an imaging system, is indicated by the blue circle for TIR-ROCS and the green circle for TIRF. The cut-off frequency for TIR-ROCS, 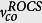 = 3.37/μm, is approximately 40% larger than in TIRF, which is 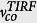 = 2.42/μm. Therefore the gain in image resolution is 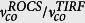 ≈ 1.39. The ratio *λ*_*ROCS*_*/λ*_*TIRF*_ = 488 nm/505 nm = 0.97 does not need to be considered, since the same laser was used for both techniques. The a-priori arbitrary choice of a 10% magnitude above the noise level does not affect the ratio of the cut-off frequencies (see Supplementary Text 5 together with Figure S4). The course of the Fourier transform indicates how well high frequency information is transferred relative to the low frequency information. This is obviously more advantageous in TIR-ROCS on the left of Fig. 3b, where the frequency spectrum *Ĩ*(*v*_*x*_, *v*_*y*_) reveals a broad yellow area. Another indication for the improved contrast and resolution is given by the line scans in Fig. 3c, which reveal significantly more pronounced intensity peaks produced by adjacent filopodia for the blue curves. Both in line 1 and line 2, the TIR-ROCS technique resolves cell structures below the resolution limit, although the distance of the peaks appear slightly increased in the image as pointed out above (*d*_*mes*_ > *d*) and in ref. 16.

### Cell dynamics observed by TIR-ROCS

**Figure 4:**
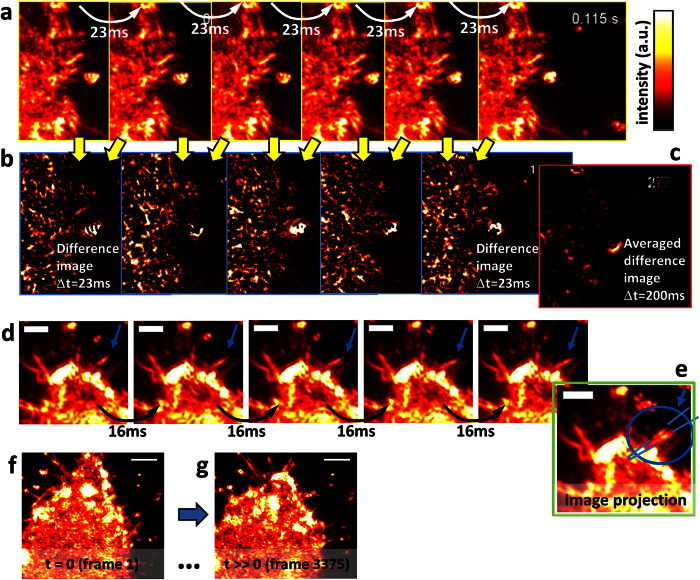
Live cell imaging at high frame rates. (**a**) Image sequence of a living J774 macrophage recorded at 44 Hz = 1/(23 ms). (**b**) Difference images of subsequent frames in (**a**). (**c**) Example of a difference image after averaging over 9 frames, corresponding to an effective frame rate of 5 Hz. (**d**) Image sequence of a dynamic filopodium of a living J774 macrophage, recorded at 63 Hz. (**e**) The image projection of (**d**) illustrates the filopodial motion. (**f**,**g**) First and last image of a TIR-ROCS series of 3375 images of a living J774 cell. Scale bars: 2 μm in (**a**–**e**) and 4 μm in (**f**,**g**).

**Figure 5:**
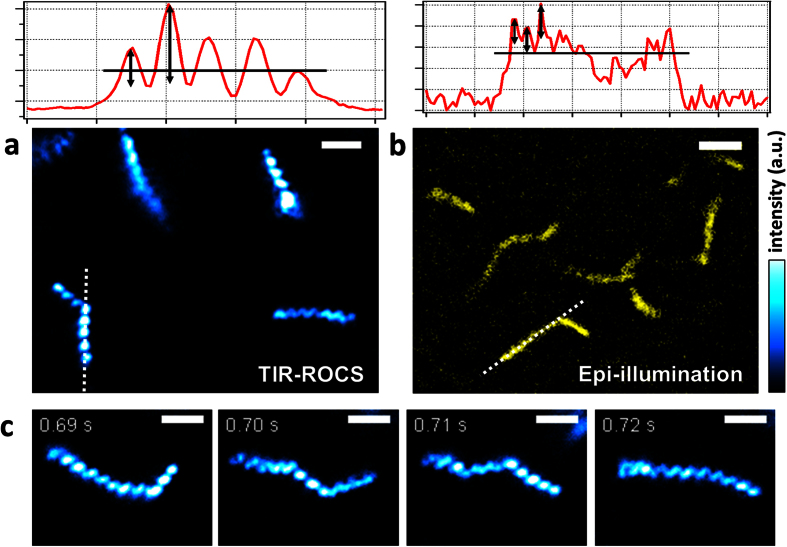
Imaging 200 nm thin, fast deforming helical bateria (Spiroplasma melliferum). (**a**) High contrast TIR-ROCS images of 4 bacteria - two in focus (bottom) and two slightly out of focus (top), (**b**) Conventional epi-illumination of several bacteria using an arc lamp shows noise and low contrast, see also the intensity line profiles along the white dotted lines in (**a**,**b**). (**c**) Image sequence of a deforming bacterium recorded at 100 Hz. Scale bars: 2 μm.

Besides the superior optical resolution and contrast, other potential advantages of TIR-ROCS are the high frame rate, no loss in image quality after many acquisitions and no post-processing. These benefits are illustrated by the Supplementary Movies 1, 2 and 3, and Figs 4 and S5 of highly active macrophages as well as Supplementary Movies 4,5 and Fig. 5 showing fast deforming helical bacteria.

Figure 4 displays two time series acquired at frame rates of 44 Hz (series a) and 63 Hz (series d), which is the operation limit of the used piezo driven scan mirror.

The time series shown in Fig. 4a acquired at 44 Hz reveals unexpectedly fast reorganization of the actin cytoskeleton of a J774 macrophage. The bright spot right to the cell is a diffusing 350 nm glass bead triggering the dynamics of nearby filopodia. The actin dynamics is well visible in Supplementary Movie 6 and in a series of difference images Fig. 4b, which display local shifts of actin filaments by the intensity structures, where red-hot colour indicates strong change and black no change. Figure 4c simulates the intensity difference observed by a fast fluorescence microscope achieving a frame rate of 1/200 ms = 5 Hz (at 150 nm spatial resolution), by averaging over 9 different images recorded at 44 Hz. Except for the bead (yellow spot), hardly any cellular dynamics can be seen (red structures).

The time series shown in Fig. 4d acquired at 63 Hz (see Supplementary Movie 2) reveals the dynamics of a filopodium (marked by a blue arrow). The angular sideward motion by a bending angle of 7° within 64 ms is indicated in the time projected image Fig. 4e, which resolves two distinct filopodium orientations, which are usually smeared out with conventional fluorescence imaging. The acquisition speed allows to distinguish between coordinated and stochastic filopodia motion, giving deeper insights into the nano-mechanical concepts of filopodia sensing (see discussion and Supplementary Text 8).

Figure 4f,g demonstrate that no loss in image quality is visible even after acquiring several thousand frames of the same macrophage. The image at time *t* = 0 (frame 1) contains the same amount of image intensity as an image acquired at *t* ≫ 0 (frame 3375), corresponding to the time period of about one minute. Both images have identical scaling and show the same specific features (filopodia).

Figure 5 displays another type of dynamic cell. The wall-less helical bacterium Spiroplasma melliferum is just 200 nm thin and undergoes fast shape changes in the form of kinks running through the helical body^25^,^26^. On the one hand only very little light is scattered at these cells, on the other hand fluorescent labelling has not been possible yet, thus making it difficult to image the dynamics of S. melliferum. Whereas Fig. 5a demonstrates the superior image quality of TIR-ROCS at only 8 ms illumination time, Fig. 5b reveals a noisy, low-contrast image obtained in epi-illumination mode using blue light from a mercury arc lamp. Figure 5c shows four subsequent images of a deforming bacterium recorded at 100 Hz revealing superior resolution and contrast.

To demonstrate the broad applicability of our method, we have additionally imaged single taxol-stabilized microtubules attached to the coverslip. Supplementary figure S8 compares the results obtained by the non-fluorescent TIR-ROCS with 25 ms acquisition time and conventional epi-fluorescence (250 ms acquisition time).

## Discussion

In this study we have shown that thousands of super-resolved, high contrast images of dynamic, unlabelled cells can be acquired at high frame rates without loss in image quality.

In particular, we could demonstrate that multiple interferences, as a consequence of coherent illumination with laser light, do not necessarily deteriorate the image quality. As illustrated in Fig. 2a, a single shot of a coherent image with illumination from only one direction does not provide any useful information. However, and this was still quite unexpected, the average over all illumination directions, i.e., the incoherent superposition of coherent images, results in a high contrast image of a complex biological structure such as an adherent J774 mouse macrophage. The high image quality, effectively free of shot noise, is based on a combination of radial coherence and tangential incoherence, such that the imaging process represents a special type of partial coherence.

The circular sweep of a focused laser beam is as fast as the beam-deflecting system, which could achieve 1,000 Hz using conventional circle scanners. In our case, the image acquisition rate was limited by the triggered camera readout at 63 Hz in a first phase and at 100 Hz after software improvements. Because of the large amount of photons backscattered from the sample, very short illumination times of a few milliseconds are sufficient, which can result in frame rates of several hundred Hertz with a fast camera.

### Super-resolution with high contrast

In our recent study^16^, we have shown by experiments, computer simulations and mathematical theory that total internal reflection rotating coherent scattering (TIR-ROCS) microscopy generates its superior resolution and contrast by interference effects. The final image is composed of a conventional incoherent image which is superposed by an interference pattern containing the high frequency information. The advantage of interferometric approaches is that the scattered field is amplified by the incident field. The superposition of the interference pattern leads to a slight local deformation of the image, which means that distances between objects appear slightly broader than they are (*d*_*mes*_ > *d*) as pointed out in Fig. 1c and revealed experimentally in Fig. 3c. Therefore, the measured distances between two adjacent intensity peaks do not define the spatial resolution of TIR-ROCS. However, the slight local image deformation will not have any consequences in most biological applications. By reducing the degree of temporal and spatial coherence, i.e., replacing the laser by a LED or even an arc-lamp, the interference pattern will be less pronounced and the light incident on the coverslip will propagate in more directions. In this case it will be technically more difficult to maintain the degree of radial (spatial) coherence and to ensure a proper TIR and dark-field mode, thus the contrast generated by the multiple local interferences will decrease, ending up in conventional dark field imaging.

### Resolution estimate

The optical resolution limit derived by Abbe^17^, defined by the wavelength *λ* and the *NA* of the lens, was originally derived for gratings. A one-dimensional grating illuminated coherently under the angle *β* reduces the resolvable minimum (half-pitch) distance *d*_*min*_ by increasing the effective numerical aperture to *NA* + *n* ∙ *sin*(*β*)^27^. Similarly, the minimum resolvable distance between two points imaged through a circular aperture can be estimated in two ways: either by using the modified Abbe formula for oblique illumination:


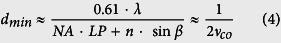


or by measuring the inverse of the frequency spectrum width 2*ν*_*co.*_ Here, *n* = 1.52 is the refractive index of the relevant embedding medium and *LP* ≈ 0.8 represents the low pass filter used for blocking the reflected light in dark-field mode. *ν*_*co*_ is the spatial cut-off frequency at 10% intensity above noise. With *NA* = 1.46, sin*β* ≈ *LP* ≈ 0.8, we find *NA*_*eff*_ = *NA LP* + *n* ∙ sin*β* = 2.46 and a theoretical resolution estimate of 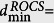 120 nm at *λ* = 0.49 μm. This value is well confirmed by analysing the radii 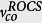
of the TIR-ROCS image spectrum shown in Fig. 3b, where we measured 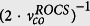 = 148 nm 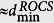 from 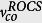 = 3.37 μm^−1^. This is compared to the TIRF mode, where 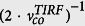 = 208 nm was measured from 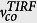 = 2.42 μm^−1^, which is also in good agreement with the theoretical value 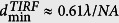 = 211 nm (where *LP* = 1 and λ ≈ 0.505 μm). Both resolution estimates for 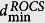 are close to the resolution measured^16^ and simulated^16^,^20^ for beads. One should remark that by using shorter wavelengths and by increasing sin*β* and *LP*, the optical resolution can be further improved.

As mentioned above, the ROCS technique is related to the imaging methods using structured illumination with either periodic gratings^23^ or highly focused image scanning microscopy (ISM)^3^. They are all based on the common physical principle that spatial frequencies on the illumination and detection side are mixed, such that the sum frequency leads to higher frequency transfer - as shown by the denominator of the resolution formula in equation (4). All techniques use oblique (i.e., modulated field) object illumination, leading to a shift of the Fourier spectrum, which results in a twofold resolution increase in the best (linear) case.

### Simplicity of the method

The charm of our technique is that no post-processing is required and the super-resolved images can be directly observed. Only a constant background interference resulting from reflections at intermediate interfaces must be recorded in advance to be subtracted from all following images, see Figure S2 in the Supplementary Information. In comparison to the technically more complicated 3D imaging method^14^ requiring intensive post-processing, the TIR-ROCS microscopy user can instantaneously decide on how to proceed in the course of his live cell experiment. Another decisive advantage is that it can be combined with fluorescence imaging techniques, where specific labelling of molecular structures reveals decisive additional information. Therefore, our approach is a high-speed variant of correlative microscopy.

### Actin cortex dynamics

Since our method generates image contrast by local refractive index changes, images remain dark in areas where the refractive index hardly changes. This is well visible in the flat growth cones in the upper part of the adherent macrophages shown in Fig. 3a. However, the small change in refractive index at the edge of the growth cone is very well visible and does not reveal any directional contrast artefacts. By inspecting the image series of Fig. 4a,b and the corresponding Supplementary Movies 7 and 8 recorded a temporal sampling of Δ*t* = 23 ms, it can be seen that strong fluctuating intensity signals propagate inward and outward relative to the cell centre. These specular patterns result from the ongoing reorganization of the actin cortex required for cell migration and responses to external disturbances such as beads in contact with the cell periphery. In Supplementary Movie 1 and in Figure S5b, directed transport of the actin cytoskeleton from the cell periphery toward the inner part of the macrophage after the binding of several beads can be clearly identified. Since the resolution of approximately 150 nm is not sufficient to resolve density variations of actin bundles and oligomers or vesicles, the image structures are partly overlaid with speckles (see also Supplementary Text 10 including Supplementary Movies 9,10,11,12). However, the local changes of the speckles provide important information about the local activity of the cell cortex and open many new possibilities to investigate cellular activity - for example in overlay with fluorescence images. A spatially resolved cell activity map can be generated by the standard deviation of all difference images. This is illustrated in the supplementary text 11 and Fig. S9 for two series recorded at 63 Hz and averaged to 6.3 Hz. The difference of both standard deviations reveals the distribution of cell activity above 7 Hz. This is not observable even with fast fluorescence imaging techniques, which are still too slow. It is therefore likely that such actin dynamics have never been observed before. On the other side, most conventional label-free techniques, such as DIC, do not provide enough contrast and resolution. The observed fast actin reorganization sheds new light on intracellular processes and opens new questions about the necessity or potential functionality of such fast structural cytoskeleton changes.

### Filopodia dynamics

From analysing the time series in Fig. 4d, we find that the filopodium bending angle of 7° within 64 ms is a very fast process, which can result from rotational Brownian motion or from active cytoskeleton and motor work. We estimated the root mean square values *θ*_*rms*_ = 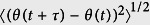

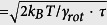
of the temporal change in orientational free diffusion for several lengths *L* between 1.8 μm … 2.2 μm and diameters *d* ranging from 0.15 μm to 0.25 μm of the filopodium^28^ to be in the range of *θ*_*rms*_ = 0.8° … 2.0°. These mean angular deflections are notably smaller than our measured value, indicating active work by actin reorganization and myosin motor proteins (see Supplementary Text 8 plus Figures S6 and S7). Whereas mechanistic models for linear filopodia elongation and retraction seem to be well accepted, models for the frequently occurring case of angular filopodia bending are still at the beginning. Understanding the nano-mechanical principles behind angular filopodia motion^29^,^30^ and sensing would provide important insights for enhanced or reduced phagocytic efficiency as an immune response mechanism for several normal or abnormal cells.

### Dynamics of helical bacteria

The time series of Fig. 5 displays shape changes, so-called kink-modes, of the only 200 nm thin wall-less helical bacterium Spiroplasma melliferum. The kinks that run along the body of the bacterium are driven by a protein chain motor^25^ and are a consequence of the successive switching and shortening of thousands of fibrin tetramers that form a contractile ribbon inside the bacterium. The dynamics and the energetics of the successive switching mechanism of the protein chain are still unknown, but could be uncovered with fast and high precision microscopy methods such as TIR-ROCS. As shown in Fig. 5, even at a 100 Hz acquisition rate the shape changes cannot be recorded smoothly and some periodic structures of the bacterium are slightly blurred within the 8 ms integration time. This indicates the fast motions and strong local torques generated by the protein chain motor, which must overcome considerable frictional forces. It is well possible that acquisition rates of several hundred Hz and with acquisition times significantly shorter than the inverse frame rate allows to uncover the molecular mechanisms of cooperative protein work, which drive the motility of these and other tiny bacteria.

### Conclusions and outlook

We have presented an imaging method which generates thousands of super-resolved, high contrast images without loss in image quality and at frame rates of 44, 63 and 100 Hertz. The technique does not require fluorophore labelling, since contrast is generated by local destructive interferences of scattered laser light. Our method is simple, since a laser beam incident under a shallow angle only needs to be scanned by 360° during one image acquisition. The laser beam reflected at the coverslip, in our case in TIR-mode, can be easily blocked by a diaphragm in the pupil plane leading to dark-field detection. Through this angular scan, and this is the most striking and unexpected result, all the multiple interferences known from imaging with laser light become practically invisible. The result is an image with very high contrast revealing small refractive index changes of the lower part of a cell in contact with the coverslip (TIR-mode).

Our technique, coherent dark field illumination and detection (TIR-ROCS), achieves a resolution of about 150 nm using a laser wavelength of 488 nm, which can be further improved by using shorter wavelengths and by circumventing the diaphragm used for dark field detection, which limits the capture angle of the scattered laser light. Although our approach of coherent, oblique illumination leads to a slight local distortion of the image, i.e., distances between two adjacent objects appear slightly larger than they are, the superior image contrast and resolution, together with the constantly high image quality over many acquisitions and the high frame rates, enable new insights into cellular dynamics.

With a faster scan mirror and a fast camera, frame rates of 200 Hz and more can easily be achieved, affording (spatial and temporal) correlation analysis of structural motions inside the cell. This includes the dynamics of vesicles, the cytoskeleton, membranes, various cell protrusions and growth cones, which can be observed with our technique by a hitherto unreached number of high contrast, super-resolved images. In this way we investigated the fast peripheral dynamics of J774 mouse macrophages, in particular unexpected fast actin reorganization, filopodia bending, growth cone motion and cargo transport as well as the fast deformations of tiny swimming bacteria and single microtubules. These observations made by TIR-ROCS open many new exciting questions in the context of nano-scale cellular mechanics. Our technique can be well combined with most fluorescence labelled imaging approaches, thus enabling correlated microscopy between specific fluorophore labelling and fast structural reorganization inside living cells.

## Additional Information

**How to cite this article**: Jünger, F. *et al*. Fast, label-free super-resolution live-cell imaging using rotating coherent scattering (ROCS) microscopy. *Sci. Rep.* **6**, 30393; doi: 10.1038/srep30393 (2016).

## Supplementary Material

Supplementary Information

Supplementary Movie 1

Supplementary Movie 2

Supplementary Movie 3

Supplementary Movie 4

Supplementary Movie 5

Supplementary Movie 6

Supplementary Movie 7

Supplementary Movie 8

Supplementary Movie 9

Supplementary Movie 10

Supplementary Movie 11

Supplementary Movie 12

## References

[b1] Kner P., Chhun B. B., Griffis E. R., Winoto L. & Gustafsson M. G. L. Super-resolution video microscopy of live cells by structured illumination. Nature Methods 6, 339–U336 (2009). 19404253 10.1038/nmeth.1324 PMC2895555

[b2] Olshausen P. V. . Superresolution Imaging of Dynamic MreB Filaments in B. subtilis - A Multiple-Motor-Driven Transport? Biophys. J. 105, 1171–1181 (2013). 24010660 10.1016/j.bpj.2013.07.038 PMC3762370

[b3] Schulz O. . Resolution doubling in fluorescence microscopy with confocal spinning-disk image scanning microscopy. Proceedings of the National Academy of Sciences 110, 21000–21005 (2013). 10.1073/pnas.1315858110 PMC3876259 24324140

[b4] Brunstein M., Hérault K. & Oheim M. Eliminating Unwanted Far-Field Excitation in Objective-Type TIRF. Part II. Combined Evanescent-Wave Excitation and Supercritical-Angle Fluorescence Detection Improves Optical Sectioning. Biophysical Journal 106, 1044–1056 (2014). 24606929 10.1016/j.bpj.2013.12.051 PMC4026779

[b5] Li D. . Extended-resolution structured illumination imaging of endocytic and cytoskeletal dynamics. Science 349, aab3500 (2015). 26315442 10.1126/science.aab3500 PMC4659358

[b6] Hell S. W. Far-Field Optical Nanoscopy. Science 316, 1153–1158 (2007). 17525330 10.1126/science.1137395

[b7] Schermelleh L., Heintzmann R. & Leonhardt H. A guide to super-resolution fluorescence microscopy. The Journal of Cell Biology 190, 165–175 (2010). 20643879 10.1083/jcb.201002018 PMC2918923

[b8] Lauterbach M. Finding defining and breaking the diffraction barrier in microscopy - a historical perspective. Optical Nanoscopy 1, 8 (2012).

[b9] Godin Antoine G., Lounis B. & Cognet L. Super-resolution Microscopy Approaches for Live Cell Imaging. Biophysical Journal 107, 1777–1784 (2014). 25418158 10.1016/j.bpj.2014.08.028 PMC4213717

[b10] Bergermann F., Alber L., Sahl S. J., Engelhardt J. & Hell S. W. 2000-fold parallelized dual-color STED fluorescence nanoscopy. Opt. Express 23, 211–223 (2015). 25835668 10.1364/OE.23.000211

[b11] Tischer C., Pralle A. & Florin E.-L. Determination and correction of position detection non-linearity in single particle tracking and three-dimensional scanning probe microscopy. Microscopy and Microanalysis 10, 1–10 (2004). 10.1017/S1431927604040140 15327703

[b12] Kress H. . Filopodia act as phagocytic tentacles and pull with discrete steps and a load-dependent velocity. Proc. Natl. Acad. Sci. 104, 11633–11638 (2007). 17620618 10.1073/pnas.0702449104 PMC1913848

[b13] Kukura P. . High-speed nanoscopic tracking of the position and orientation of a single virus. Nature Methods 6, 923–927 (2009). 19881510 10.1038/nmeth.1395

[b14] Cotte Y. . Marker-free phase nanoscopy. Nature Photonics 7, 113–117 (2013).

[b15] Cronin D. J. & Smith A. E. Dynamic Coherent Optical System. OPTICE 12, 120250–120250 (1973).

[b16] v. Olshausen P. & Rohrbach A. Coherent Total Internal Reflection Dark Field Microscopy - an approach to label-free imaging beyond the diffraction limit. Opt. Lett. 38, 4066–4069 (2013). 24321924 10.1364/OL.38.004066

[b17] Abbe E. Beiträge zur Theorie des Mikroskops und der mikroskopischen Wahrnehmung. Archiv f. mikrosk. Anatomie 9, 413–418 (1873).

[b18] Dan D. . DMD-based LED-illumination Super-resolution and optical sectioning microscopy. Sci Rep-Uk 3 (2013). 10.1038/srep01116 PMC3552285 23346373

[b19] Zheng G., Horstmeyer R. & Yang C. Wide-field, high-resolution Fourier ptychographic microscopy. Nature Photonics 7, 739–745 (2013). 25243016 10.1038/nphoton.2013.187 PMC4169052

[b20] Wicker K. & Heintzmann R. Resolving a misconception about structured illumination. Nature Photonics 8, 342–344 (2014).

[b21] Lukosz W. Optical Systems with Resolving Powers Exceeding the Classical Limit. II. J. Opt. Soc. Am. 57, 932–939 (1967).

[b22] Gustafsson M. G. L., Agard D. A. & Sedat & (IM)-M-5: 3D widefield light microscopy with better than 100 nm axial resolution. Journal of Microscopy-Oxford 195, 10–16 (1999). 10.1046/j.1365-2818.1999.00576.x 10444297

[b23] Heintzmann R. & Cremer C. G. In BiOS Europe'98 185–196 (International Society for Optics and Photonics, 1999).

[b24] Bohren C. F. & Huffman D. R. Absorption and scattering of light by small particles. (John Wiley & Sons, 2004).

[b25] Trachtenberg S. The cytoskeleton of Spiroplasma: A complex linear motor. Journal of Molecular Microbiology and Biotechnology 11, 265–283 (2006). 16983201 10.1159/000094060

[b26] Koch M. & Rohrbach A. Object adapted optical trapping and shape tracking of energy switching helical bacteria. Nature Photonics 6, 680–686 (2012).

[b27] Singer W., Totzeck M. & Gross H. Physical Image formation, Vol. 2. (Wiley-VCH, Weinheim, 2005).

[b28] Mattila P. K. & Lappalainen P. Filopodia: molecular architecture and cellular functions. Nat Rev Mol Cell Biol 9, 446–454 (2008). 18464790 10.1038/nrm2406

[b29] Oldenbourg R., Katoh K. & Danuser G. Mechanism of lateral movement of filopodia and radial actin bundles across neuronal growth cones. Biophysical Journal 78, 1176–1182 (2000). 10692307 10.1016/S0006-3495(00)76675-6 PMC1300720

[b30] Bornschlögl T. How filopodia pull: What we know about the mechanics and dynamics of filopodia. Cytoskeleton 70, 590–603 (2013). 23959922 10.1002/cm.21130

