# Supplementary material for: Fast, label-free super-resolution live-cell imaging using rotating coherent scattering (ROCS) microscopy
Source: Sci Rep. 2016 Jul 28;6:30393. doi: 10.1038/srep30393 (PMC4964612; doi:10.1038/srep30393)
Supplement: Supplementary Information [file srep30393-s1.pdf]

Supplementary Information to

Fast, label-free super-resolution live-cell imaging using rotating coherent scattering (ROCS) microscopy

Felix Jünger[1], Philipp v. Olshausen[1,2] and Alexander Rohrbach[1,3]

[1] Laboratory for Bio- and Nano-Photonics, Department of Microsystems Engineering, University of Freiburg, Germany

[2] Testo AG, Testo-Straße 1, 79853 Lenzkirch, Germany

[3] BIOSS Centre for Biological Signalling Studies, University of Freiburg, Germany

Supplementary Text 1: Experimental Setup

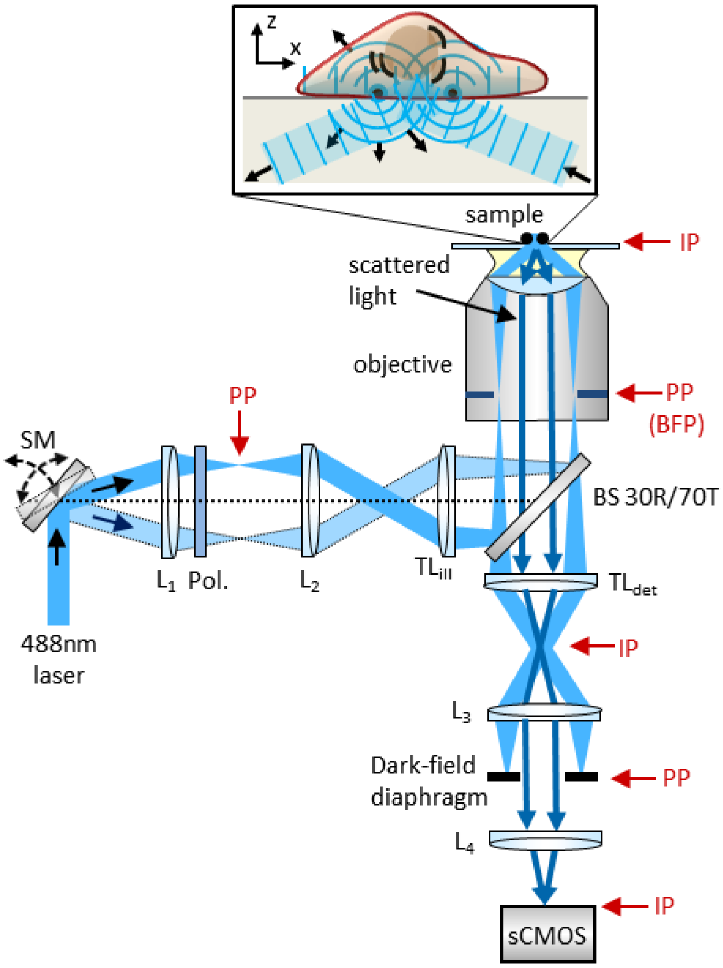

Figure S1 | Experimental setup. A schematic overview of the experimental TIR-ROCS microscope setup used in this study. Light blue beams indicate the course of the incident λ_0 = 488 nm laser beam, which is deflected in two dimensions perpendicular to the optical axis (dotted black line) by the scan mirror (SM), as indicated by the second, semi-transparent light blue beam emanating from the SM. The light scattered at the cell has the same wavelength λ_0, but is displayed in a darker blue for better visibility. The upper box illustrates the evanescent illumination of and scattering at the cell sample, where solid blue lines indicate wavefronts of the coherent illumination light and the scattered light, respectively. The image plane and its conjugate planes are indicated by red arrows labelled "IP", whereas "PP" denotes pupil planes. Further details of the optical components are given in the text.

Sample illumination

The experimental setup is shown in Figure S1. A collimated, linearly polarized 488 nm laser beam (2214-20SL, JDSU, Milpitas, CA, USA) is deflected by a two-axis tip-tilt scanning mirror (PI S-330.4SL, Physik Instrumente, Karlsruhe, Germany). An acousto-optical tuneable filter (AA.AOTF.4TC, AA Opto-Electronic, Orsay, France) placed at the beginning of the beam path assures that the laser power is kept constant at P = 5.0 mW. A 20x beam expander (S6ASS2420-121, Sill Optics GmbH, Wendelstein, Germany) widens the beam diameter to 14 mm before hitting the scan mirror (AOTF and beam expander not shown in Figure S1). The scan mirror, which was optimized for a very low dither, allows deflecting the beam by an angle of 2.1 mrad at a circular frequency of up to 63 Hz. In a later stage, this scan mirror was replaced by another two-axis steerable mirror (Newport FSM-200, Newport Spectra-Physics GmbH, Darmstadt, Germany), which achieved a similar deflection at >100 Hz. The lens L_1 (Qioptiq SAS, Paris, France) with f_1 = 400 mm focuses the beam

into the pupil plane (PP). A half-wave plate in combination with an S-waveplate (Pol., Mountain Photonics GmbH, Landsberg am Lech, Germany) is used to assure azimuth polarization for all illumination directions. A 4f system consisting of an achromatic lens L_2 with f_2 = 120 mm and a tube lens TL_{ill} with f_{TL} = 200 mm (Thorlabs, Newton, NJ, USA) installed in the microscope's rear port (Leica DM IRBE, Leica, Germany) focuses the beam into the outermost ring of the back focal plane (BFP) of an objective lens (HCX PL Apo, 100×, NA 1.46, oil immersion, Leica Microsystems, Wetzlar, Germany) with high *NA*, resulting in a plane wave emanating from the objective lens under such a high angle that it undergoes TIR at the interface between the glass coverslip (n_i = 1.52) and the sample in watery solution (n_t = 1.33). The resulting evanescent field illuminates only a thin section of the cell with a nominal penetration depth of roughly 70 nm. Displacing the beam from the optical axis by the scan mirror changes the azimuthal direction ϕ of the evanescent wave illumination in the sample plane (image plane, IP).

Detection beam path

The reflected light as well as some of the light scattered by the sample is collected by the objective lens. A 30/70 beam splitter (AHF Analysentechnik AG, Tübingen, Germany) transmits 70% of this light to the detection beam path, consisting of two f = 160 mm achromat lenses (TL_{det}, L_3; Thorlabs). To constitute dark-field detection, a diaphragm is placed in a PP that blocks all TIR light. Only the scattered light passes the diaphragm and the lens L_4 and forms an image on a sCMOS camera (ORCA-Flash4.0 V2, Hamamatsu Photonics, Hamamatsu, Japan), which allows image acquisition at frame rates of 100 Hz in our software environment . In case of fluorescently labelled cells, an additional emission filter (550/88, Semrock, Lake Forest, IL, USA; not shown) is placed in the detection beam path to suppress any remaining excitation light transmitted by the beam splitter, which carries much more intensity and would otherwise outshine the fluorescence signal.

Supplementary Text 2: Background subtraction

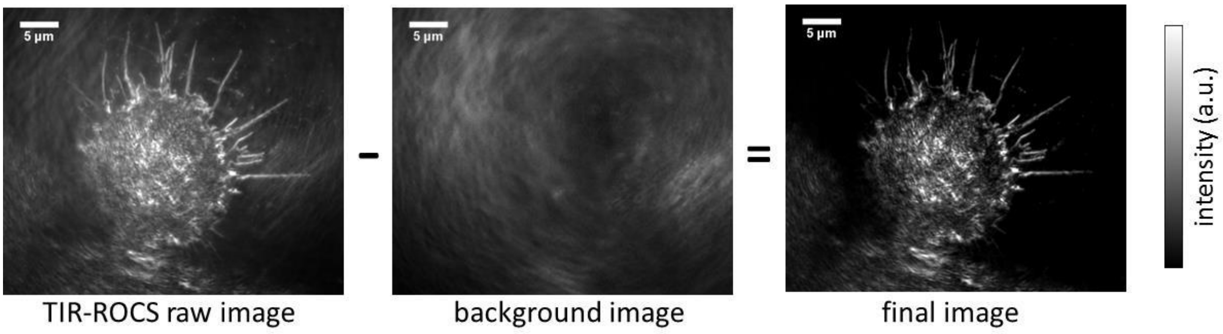

Figure S2 | Principle of background subtraction. A separate background image can be subtracted from the raw ROCS image to obtain a sharp final image.

A constant background interference, mainly resulting from reflections at interfaces of the microscope's optical components, is superimposed to every raw TIR-ROCS image. To get rid of those back-reflection patterns, a separate background image must be recorded to be subtracted from all raw images as illustrated by Figure S2. The background image is obtained by moving the cell sample aside, i.e., out of the camera ROI, by means of a movable microscope stage without altering the *z* position of the microscope objective or the height of the sample chamber containing the cell. Then, a second TIR-ROCS image with identical parameters and full 2π turn is acquired, containing only the pattern from the reflections of the optical interfaces. Since the raw image can be considered as an incoherent superposition of the mere cell image and the background image, a simple subtraction of the background from the raw image reveals a clear, reflection-corrected final image, in which cell structures become distinguishable much more clearly. Background subtraction has been applied to all images shown in Figures 3-5 and also Supplementary Movies 1-9 and 11.

Supplementary Text 3: Selection of spatial frequencies under oblique illumination

The spectrum of the total electric field is the sum of the totally internal reflected field and scattered field spectra $\tilde{E}_r(\mathbf{k}) + \tilde{E}_s(\mathbf{k}) = \tilde{E}_r(\mathbf{k}) + E_i \cdot F(\mathbf{k} - \mathbf{k_i}) \cdot OTF_A(\mathbf{k})$. In dark-field imaging, the unscattered reflected field $\tilde{E}_r(\mathbf{k})$ is blocked by a diaphragm, reducing the effective detection numerical aperture by the factor LP = 0.8.

The following construction using Ewald spheres illustrates the high optical transfer of high spatial object frequencies, which are cut out of the object spectrum $F(\mathbf{k}) = FT[f(\mathbf{r})]$ by the coherent optical transfer function $OTF_A(\mathbf{k})$ with a width of $2k_0 \cdot NA_{det}$ (Ewald sphere segment shown in red). The object was assumed to be two point scatterers $f(x,z) = \delta(x + \frac{dx}{2}) + \delta(x - \frac{dx}{2})$ in a distance dx to each other such that the object spectrum is $F(\mathbf{g} = \mathbf{k} - \mathbf{k_i}) = 2\cos\left(k_x \frac{dx}{2} - k_{ix} \frac{dx}{2}\right)$, which is illustrated by the green background.

The selected spectrum of the scattered field $\tilde{E}_s(k_x)$ is shown at the bottom for normal incidence (in blue) and oblique incidence (in red) together with the reflected plane wave (read peak on the left), which is not transmitted through the diaphragm. For oblique incidence the spectrum $\tilde{E}_s(k_x)$ shows a significantly higher amplitude at higher frequencies.

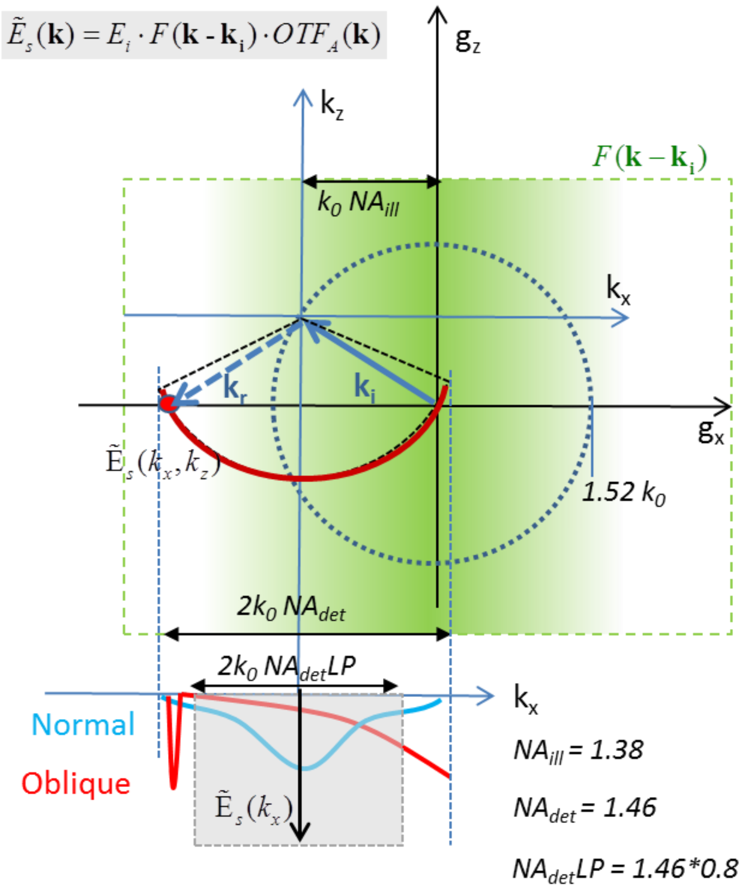

Figure S3 | Scheme to illustrate the selection of spatial frequencies under oblique illumination.

Supplementary Text 4: Cell preparation

All cells were cultured at 37°C in an atmosphere containing 5% CO_2. During growth and experiments, the cells were suspended in cell medium (Dulbecco's Modified Eagle Medium (DMEM) GlutaMAX, Invitrogen Life Technologies, Carlsbad, CA) with 10% fetal bovine serum added). Prior to each experiment, the cells were detached from the bottom of the cell culture flask and transferred onto a microscope cover glass (ibidi µ-Dish No. 1.5H (170 µm +/- 5 µm) with glass bottom, ibidi GmbH, Martinsried, Germany).

One benefit of the ROCS technique is that it requires no object staining whatsoever. The cells used for the experiments shown in Figures 4 and 5 did not receive any additional treatment. However, to acquire conventional TIRF images for comparison (see Figure 3), the cells have been fixed and stained prior to the experiments. The fixation procedure includes removing the cell suspension medium, washing the cells three times in phosphate buffered saline (PBS) without supplements (Biochrom GmbH, Berlin, Germany), incubating the cells for 15 minutes at T = 37°C in 2.5 ml 4% paraformaldehyde (PFA) solution, which is then replaced by 2.5 ml PBS at 37°C right before the experiments. Membrane staining is achieved by first washing the cells three times in PBS without supplements, then incubating the cells at 37°C in 2.5 ml WGA-FITC, diluted 1:500 in PBS solution. After letting the WGA-FITC solution take effect for 10 minutes, cells are again washed three times in PBS and then transferred to the microscope. The cells shown in Figure 2 were fixed before image acquisition but did not receive membrane staining.

Supplementary Text 5: Analysis of image spectra

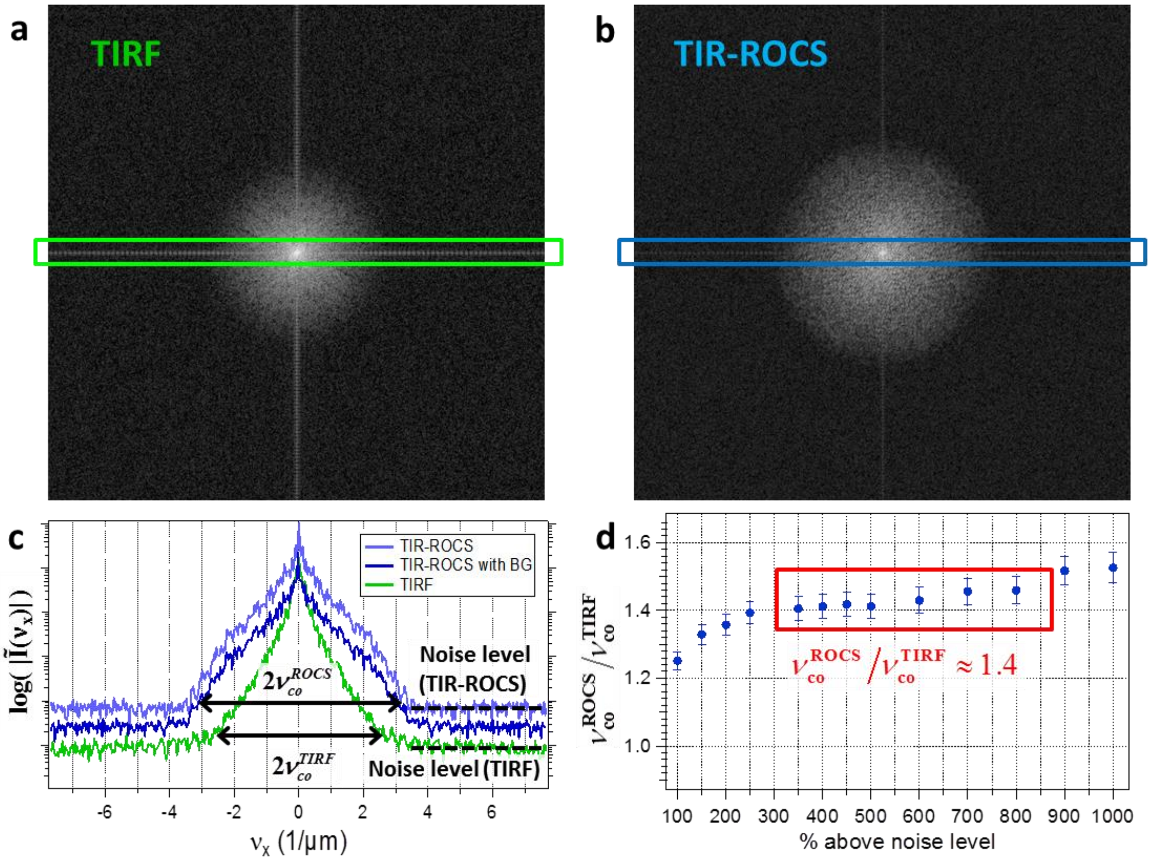

Figure S4 | Resolution estimation by image spectra. Logarithmic grayscale representation of image spectra $\tilde{I}(\nu_x,\nu_y)$ for TIRF **(a)** and TIR-ROCS **(b)**. **(c)** Averaged intensity line scans $\tilde{I}(\nu_x,0)$ for both imaging modes revealing the maximal transferred spatial frequencies ν_{co} above noise. The spectral cut-off diameters $2\nu_{co}$ are indicated by black arrows. **(d)** The ratio between both cut-off frequencies $\nu_{co}^{ROCS}/\nu_{co}^{TIRF} \approx 1.4$ remains constant for a broad range of levels above noise.

Contrast and resolution in both images $I(x,y)$ from the same cell (Figure 3a in the main text) are compared globally by the modulus of the Fourier transform $\tilde{I}(\nu_x,\nu_y)$ (Figure 3b). While the exposure time in TIR-ROCS mode was merely 20 ms, TIRF images were taken with 5 seconds integration time to achieve a comparable image contrast. Because of the long integration time for TIRF, the signal to noise ratio $SNR = I/\sqrt{I}$ is better than in TIR-ROCS.

The Fourier spectra $\tilde{I}(\nu_x,\nu_y)$ of the images were calculated with ImageJ (http://rsbweb.nih.gov/ij/). Note that all spectra (Figure S4a,b) are displayed logarithmically and with 8 bit information depth, such that all intensity information $\tilde{I}(\nu_x,\nu_y)$ is logarithmically distributed between 0 and 255 units.

Diameter of the image spectrum

The width of the circular shaped image spectrum $\log\left(\tilde{I}(\nu_x,\nu_y)\right)$ (see Figure S4a,b) can be used to estimate the resolution achieved by an optical system (see discussion following Figure 3 in the main text). This width is quantified by an a-priori arbitrarily chosen corner frequency ν_{co}, which corresponds to a defined magnitude $\log\left(\tilde{I}(\nu_x=\nu_{co},\nu_y=\nu_{co})\right)=\log\left(\tilde{I}_{Noise}\right)\cdot\left(1+\frac{N}{100}\right)$, which is N percent above the constant noise level $\tilde{I}_{Noise}$. The latter is different for TIRF and TIR-ROCS (Figure S4c).

The green line profile in Figure S4c corresponds to a TIRF image, whereas the light and dark blue lines correspond to TIR-ROCS image mode with and without applied background subtraction (see

Figure S2), respectively. The line profiles are taken along the ν_x axes as denoted by the boxes in Figure S4a and S4b with averaging over the height $\Delta\nu_y$ of the boxes. Line profiles along ν_y would yield basically the same results due to an approximately rotational symmetry of $\tilde{I}(\nu_x,\nu_y)$. As visible in Figure S4c, the background subtraction (light blue line) hardly changes the shape of the spectrum (removal of low frequencies around $\tilde{I}(0,0)$), thus increasing the overall offset. Figure S4d demonstrates that the ratio of cut-off frequencies for both imaging modes, $\nu_{co}^{ROCS} / \nu_{co}^{TIRF}$ ≈ 1.4, does not change significantly for a broad range of reasonably chosen levels above noise.

Supplementary Text 6: TIR-ROCS movies of J774 macrophages and helical bacteria

A brief description of the Supplementary Movies 1 – 5 is given in the following.

Supplementary Movie 1: An untreated, living macrophage, interacting with 2*R* = 350 nm SiO_2 beads, was imaged at 63 Hz for several minutes. The data presented in Figure S5 originate from this movie. The movie is color-coded with the ‘Cyan Hot’ lookup table of the public domain software ImageJ.

Supplementary Movie 2: An untreated, living macrophage, interacting with 2*R* = 350 nm SiO_2 beads, was imaged at 63 Hz for several minutes. The data presented in Figure 4d were extracted from this movie and the motion of one prominent filopodium was analysed and discussed in the main text and in Supplementary Text 8.

Supplementary Movie 3: An untreated, living macrophage was imaged at 44 Hz for several minutes. 2*R* = 350 nm SiO_2 beads were added to the sample chamber containing the cell right before acquisition started. After some seconds, the beads sink to the bottom of the sample chamber due to segregation, thus becoming visible in TIR-ROCS microscopy. The ongoing activity of the cellular growth cone and filopodia is visible throughout the whole movie. Some beads are eventually bound to filopodia initiating transport processes.

Supplementary Movie 4: Several fast moving helical bacteria Spiroplasma melliferum are imaged at the same time with 100 Hz. These bacteria can actively propagate by fast shape changes in form of kinks propagating through the bacterial body. The helical shape of those bacteria close to the coverslip can be clearly resolved with TIR-ROCS imaging, whereas the image gets blurred when bacteria move out of focus, i.e., away from the coverslip.

Supplementary Movie 5: A single helical bacterium is imaged at 100 Hz and displayed with 10x slow motion, revealing a kink running through the bacterium from the lower left end to the upper right within a very short time in the range of 100 milliseconds.

Supplementary Text 7 - Transport along filopodia and growth cones

Cell biology and biophysics is one particularly important, promising and well suitable area of application for TIR-ROCS microscopy. The observation of some examples of cellular processes have been demonstrated in this study. A great advantage of our method is that it can generate super-resolution images with constantly good image contrast over minutes of continuous acquisition, allowing for the study of long-term cellular processes. In Figure S5a, two regions of interest are further investigated by three image sequences. The super-resolved, high contrast images show a dynamic J774 macrophage, which is rich of filopodia. Diffusing 350 nm glass (SiO_2) beads are bound randomly to the periphery of the macrophage and one of three beads is transported towards the cell interior (see yellow arrow in Figure S5b). At the same time a cellular growth cone propagates in the opposite direction at high velocity (see green arrow). The time series shown in Figure S5c,d from a different ROI reveals a similar behaviour: the bead is transported along an adherent filopodium towards the cell (see blue arrow), while the growth cone propagates towards the approaching bead (see light grey arrow). In the Supplementary Movie 1 and in Figure S5b,c it can be seen that the overall amount of image intensity, i.e., the amount of scattered light is not reducing with time.

Figure S5f,g show a magnification of the region highlighted in the last panel of Fig. S5d (white box). The 350 nm silica bead is transported at high velocity along the growth cone towards the cell interior. The sum of the 5 images from (f) is shown in Figure S5g, where the projected bead trajectory of 0.432 µm length within 4×16 ms results in a very high transport speed of v = 0.432µm/64ms = 6.75µm/s.

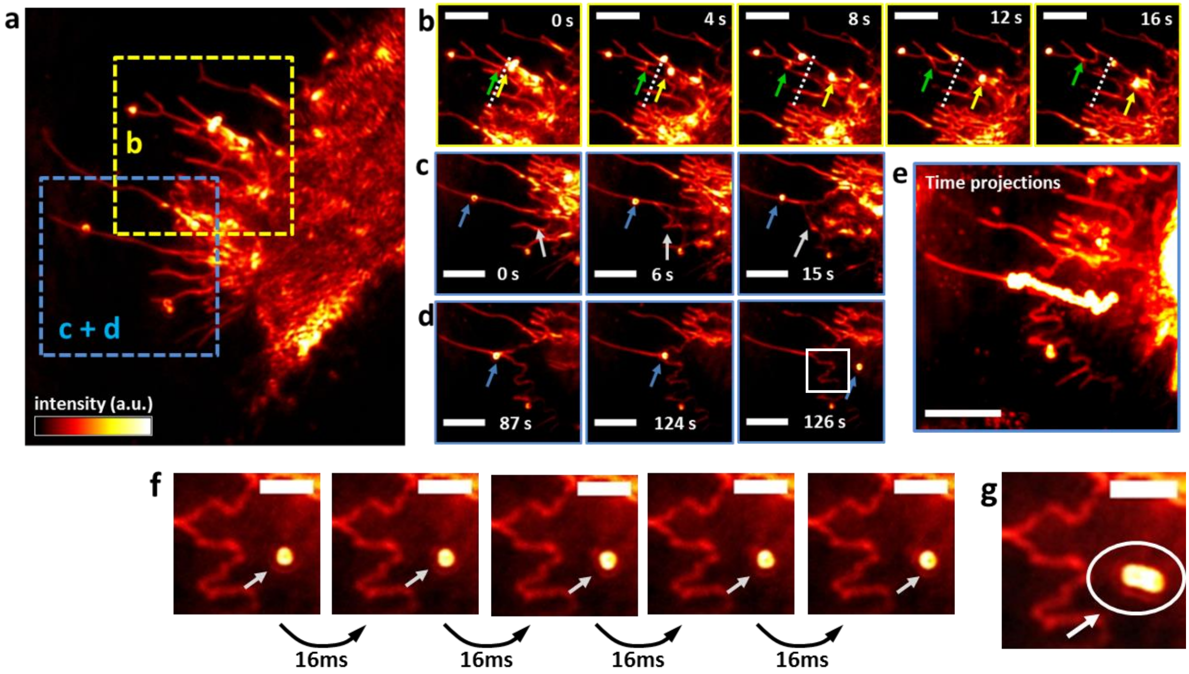

Figure S5 | Live cell imaging at high frame rates. (a) Image sequence recorded at 63 Hz = 1/(16ms) showing a 350 nm glass bead, transported along a cellular growth cone. **(b)** The propagation of a growth cone to the left (indicated by green arrows) and simultaneous retraction of a 350 nm glass bead to the right (indicated by yellow arrows). The dotted white line is placed at the start position and serves as a guide to the eye to visualize the motion. **(c)** Propagation of a growth cone (light grey arrows) and directed transport of a bead along an adherent filopodium (light blue arrows). **(d)** Transport of the same glass bead at high velocity indicated by blue arrows. (e) The trace of the bead transport is visualized as a time projection, which is a straight extension of the filopodium inwards the cell. Scale bars: 4 µm. **(f)** Image sequence recorded at 63 Hz = 1/(16ms) showing a 350 nm glass bead, transported along a cellular growth cone. **(g)** The image projection of (f) illustrates the fast transport of the bead at $v \approx$ 6.75µm/s. Scale bar: 2µm.

It has been shown recently [2] that 1 µm latex beads are transported discontinuously along adherent filopodia of J774 macrophages with velocities of up to 120 nm/s. Here, the bead appeared to be connected to the actin backbone inside the filopodium through transmembrane proteins. The backbone is shifted backwards by myosin II motors [3, 4], which maintain the retrograde flow of the backbone. In our present study, we could observe that 0.35 µm glass beads are transported by 1.1-3.2 fold higher velocity of 135-390 nm/s along the filopodium (Figure S5c) and with an extraordinarily high velocity of 6.75 µm/s along the growth cones (Figure S5d). The mechanisms controlling this velocity - such as the friction of the bead in contact with the membrane or inside the actin cortex - are unclear, but may be highly relevant to uncover cellular principles of immune efficiency. The time projection of the straight bead trajectory Figure S5e indicates that the backbone and the retrograde flow reach far into the cell interior, before the bead is disconnected. Evenly interesting is that the edges of the growth cones propagate in the direction opposite to the bead transport (Figure S5b). Whereas growth cone propagation results from actin polymerization at the end of the backbones, the backbones with the beads attached are retracted by myosin II motors [3-5]. The data provided by TIR-ROCS suggest that growth cones propagate in the direction of resting beads to increase the efficiency of phagocytic uptake. However, further investigations are required to strengthen this hypothesis.

Supplementary Text 8: Fast angular motion of filopodia seems to be driven by active processes

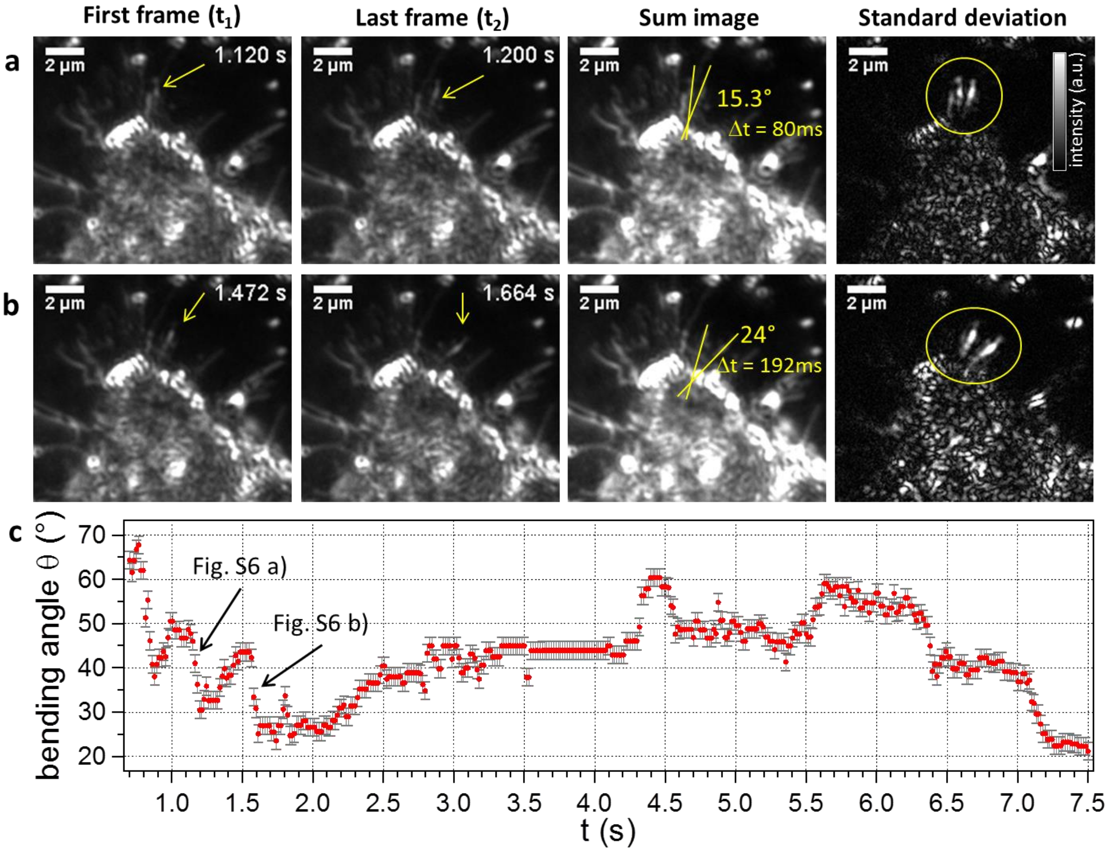

Figure S6 | J774 macrophage with filopodia handling $2R$ = 350 nm SiO_2 beads. (a) (b) First two columns show snapshots from a TIR-ROCS movie recorded with 63 Hz, yellow arrows indicate a filopodium whose motion is analysed. Third column shows the sum of the images in the first columns. The initial and final orientation of the filopodium under investigation is marked by yellow lines together with the angle between both orientations as measured with the ImageJ angle tool. Column 4 shows the standard deviation of images 1 and 2 to better visualize the movement of the filopodium. Rows (a) and (b) show the same filopodium at different times. **(c)** Trajectory of the bending angle $\theta(t)$ for the filopodium seen in (a) and (b) and in Supplementary Movie 2. Data points are shown in red, grey error bars correspond to a reading error $\Delta\theta$=2°.

From Supplementary Movie 2, the motion of a filopodium was analysed as exemplarily shown in Figure 4d in the main text. The measured bending angles were compared to the calculated root mean square values $\theta_{rms} = \left\langle \left(\theta(t+\tau)-\theta(t)\right)^2 \right\rangle^{1/2} = \sqrt{2k_BT/\gamma_{rot}\cdot\tau}$ of the temporal change in orientational free diffusion of the filopodium. For a mathematical description of angular filopodial motion, the filopodium was modelled as a cylinder with length L and diameter D. The viscous drag of a cylinder that performs a rotational motion perpendicular to the cylinder axis can be expressed as $\gamma_{rot} = \frac{\pi\cdot\eta\cdot(L+2\xi)^3}{3\left(\ln(L/D)+\delta_{rot}\right)}\cdot q(z)$ [6], where η is the viscosity of the surrounding medium and $q(z=D)\approx 2$ is a z- distance dependent correction factor accounting for the increase in viscous drag in front of a planar interface (glass coverslip). ξ is the displacement of the pivot from the cylinder's centre of mass, i.e., $\xi = L/2$ in our case, and δ_{rot} = -0.722 is an effective end correction [7]. With the knowledge of γ_{rot}, the rotational mean squared displacement $\left\langle \left(\theta(t+\tau)-\theta(t)\right)^2 \right\rangle = 2k_BT/\gamma_{rot}\cdot\tau$ for free diffusion was calculated and compared to the findings in the results section.

Assuming the length to be between L = 1.8 µm … 2.2 µm and the diameter d ranging from 0.15 µm to 0.25 µm, we calculated the bending angle to be in the range of θ_{rms} = 0.8° … 2.0°. Out of six distinct angular movements of the same filopodium, four measurements are far from the estimated free angular diffusion marked by the red region in Figure S7, giving rise to the assumption that these motions are

actively driven. In between the time periods analysed, the filopodium was in rest or performed only small diffusive movements that are not resolvable in the movie (see Figure S6c and Supplementary Movie 2).

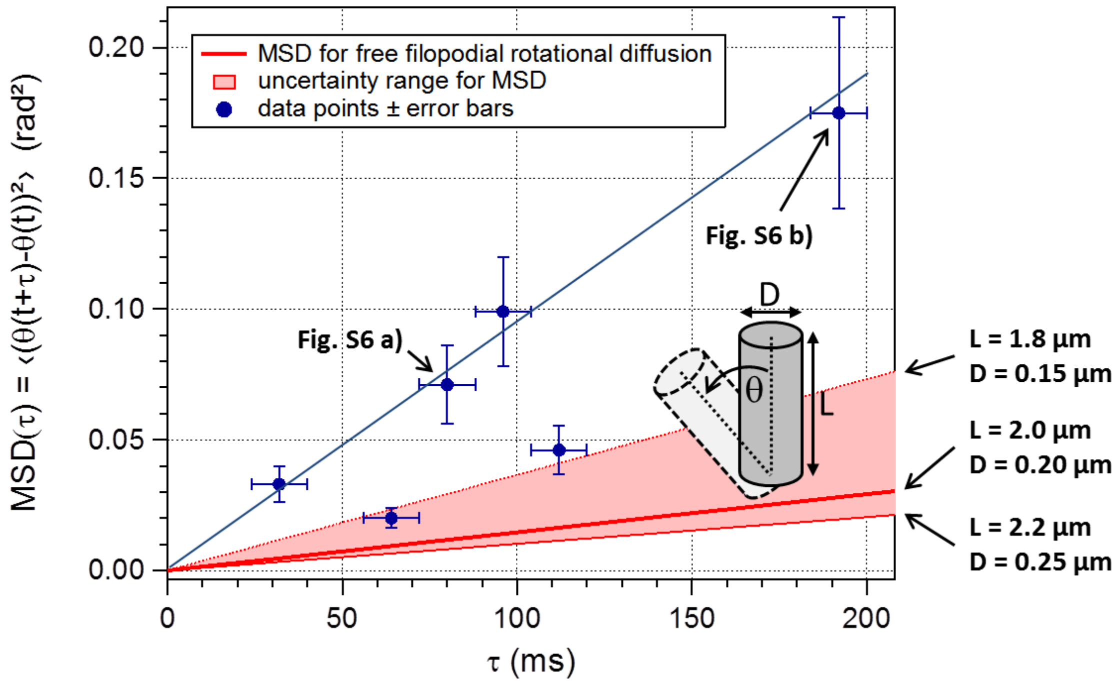

Figure S7 | Mean square displacement of angular deflection $\theta(t)$ (in rad). The red line and the red shaded region indicate possible MSD values for filopodia with lengths L ranging from 1.8 µm to 2.2 µm and thicknesses D ranging from 150 nm to 250 nm. The blue line connecting four data points that are outside the red region serves as a guide to the eye. Blue circles denote MSD(τ) values calculated from the measurement data. Error bars correspond to $\Delta\theta = 2°$ reading error from the images and $\Delta\tau = 8$ ms due to discrete temporal sampling with $\tau_{exp} = 16$ ms.

Supplementary Text 9: TIR-ROCS imaging of microtubules

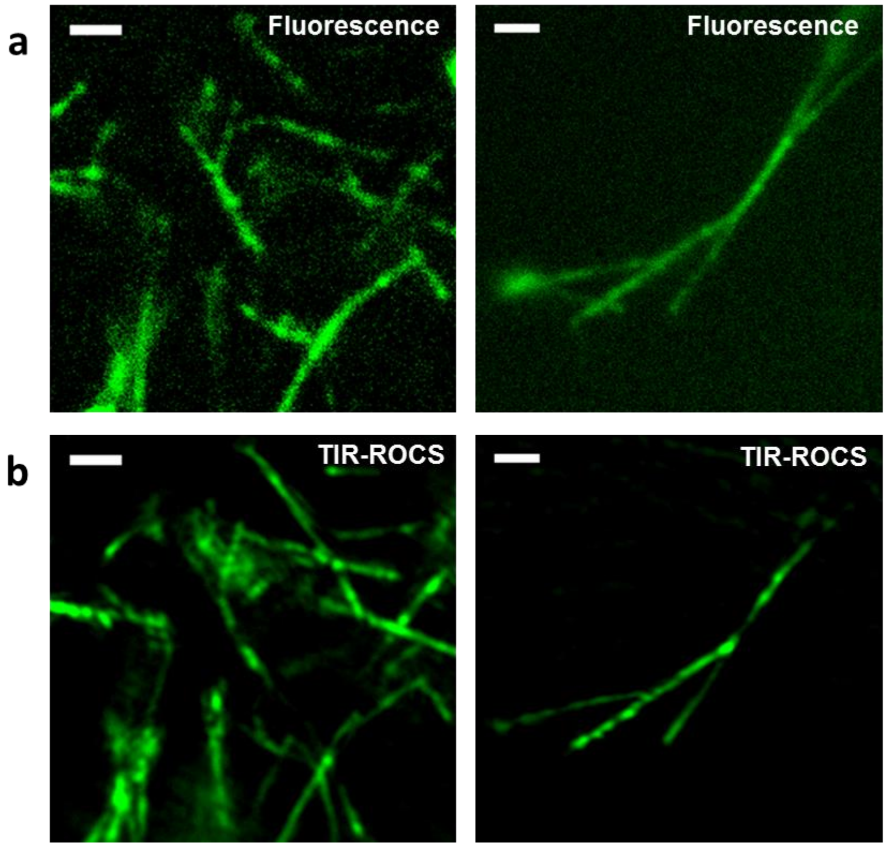

Figure S8 | Images of Taxol-stabilized and fluorescently labelled microtubules. (a) Fluorescence images acquired with 250 ms exposure time, **(b)** TIR-ROCS images taken with 25 ms exposure time. No fluorescence excitation is required to acquire the TIR-ROCS images. Scale bars are 2 µm in length.

Microtubules (MT) are components of the eukaryotic cytoskeleton which form dynamic filamentous structures with several tens of micrometres in length. Their diameter is only 25 nm, which makes it very difficult to image single microtubules via conventional light microscopy. Fluorescence imaging of labelled MT has become the standard method despite the known drawbacks of rapid photobleaching. The ability of TIR-ROCS to image changes in refractive index allows the label-free imaging of MT filaments without photobleaching and superior image contrast compared to fluorescence microscopy. Figure S8 displays a comparison of epi-fluorescence images (a) and TIR-ROCS images (b) of MTs attached to the microscope's coverslip forming network-like or branched structures. MTs were stabilized by addition of taxol, which prevents filament depolymerisation. Note that the integration time of TIR-ROCS was 10 times smaller (25 ms) as opposed to the fluorescence mode (250 ms), yet revealing a better image quality in terms of higher contrast and strongly reduced background.

Supplementary Text 10: Dynamic speckle patterns stem from cell activity

To rule out that that the dynamic speckle patterns seen in all TIR-ROCS movies of living macrophages (see Suppl. Movies 1-3, 7-9 and 11) arise from artefacts caused by the optical components in the beam path, we compared the resulting movies for a fixed cell (Movies 11 and 12) and a living, untreated cell (Movies 9 and 10). In addition to movies in TIR-ROCS mode, where one camera image consists of the averaged intensity over one full round trip of the sweeping laser focus, movies with illumination under one fixed angle, i.e., with scan mirror in rest, were acquired. Exposure time for each movie 9-12 was 50 ms with a camera frame rate of 20 Hz.

Supplementary Movie 9: untreated J774 cell, TIR-ROCS illumination
Supplementary Movie 10: untreated J774 cell, illumination under one fixed illumination angle
Supplementary Movie 11: fixed J774 cell, TIR-ROCS illumination
Supplementary Movie 12: fixed J774 cell, illumination under one fixed illumination angle

Comparing the dynamic speckle motion in Movies 9 and 10, no difference in speckle dynamics can be seen, no matter whether integration over one round trip (TIR-ROCS) is performed or the sample is illuminated only from one side. Note that speckle movement can only be observed in the region of cell interior, which can best be seen in TIR-ROCS mode (Supplementary Movie 9), since this acquisition is mostly free of coherent imaging artefacts as explained in the main text. Comparing these findings to Movies 11 and 12, where no speckle movement can be observed, gives rise to the conclusion that the speckle activity is caused by ongoing processes of structural changes, movement and reorganization within the interior of a living cell. The detailed analysis of the speckle dynamics opens the opportunity of detailed examinations of ongoing cellular processes, which, however, requires further investigations and is beyond the scope of this study.

Supplementary Text 11 - Spatially resolved cytoskeleton activity

A spatially resolved cell activity map can be generated by the standard deviation of all difference images from an image series. Black areas in the difference images indicate no changes in the image, bright areas indicate strong changes. The standard deviation over all difference images is the larger, the stronger the intensity changes from one image to the next. This activity mapping is illustrated in Figure S9 for two series recorded at 63 Hz and averaged to 6.3 Hz. The difference of both standard deviations reveals the distribution of cell activity above 7 Hz.

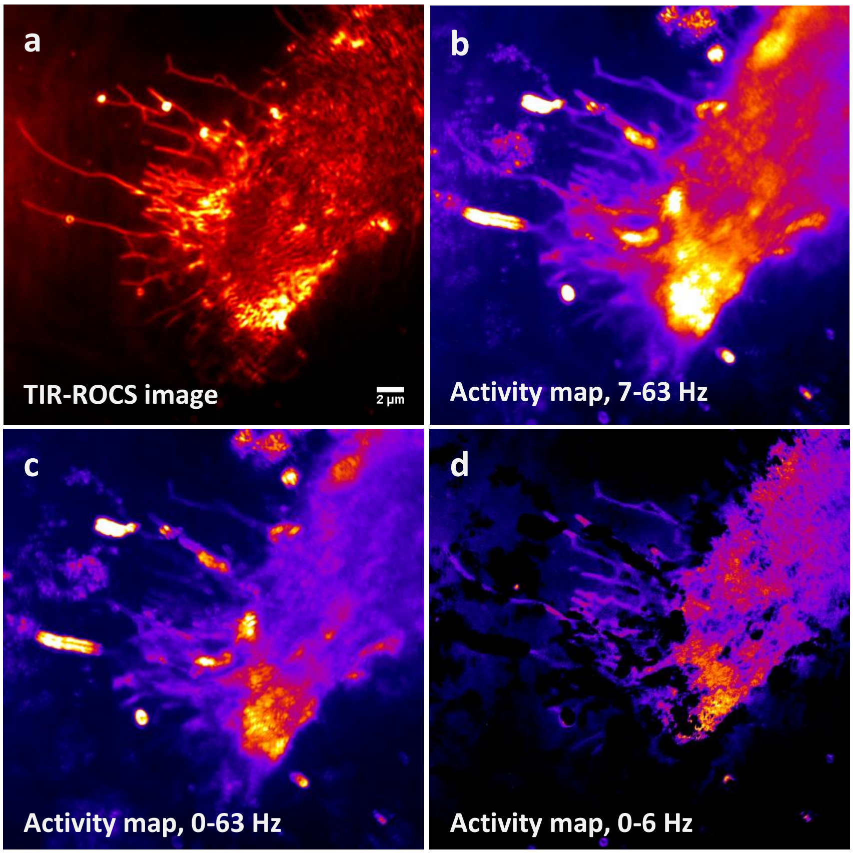

Figure S9 | **Spatially resolved activity maps of a living macrophage**. **(a)** Single TIR-ROCS image with a spatial resolution of 150 nm. **(b)** Map of cellular activity between 7 Hz and 63 Hz. **(c,d)** Standard deviations from a series of difference images recorded at 63 Hz and 6.3 Hz. The difference of both standard deviations generates the activity map between 7 Hz and 63 Hz shown in (b).

Supplementary Text 12: Comparison to reflectance interference contrast microscopy (RICM)

The RICM technique is old and well-established. It is based on the interference of mostly planar waves under normal incidence that are reflected from the cell surface and the glass interface. Due to the very short coherence length of the partially coherent light source (e.g., single emission lines from a Mercury arc lamp) the background can be well suppressed.

We have tested the RICM technique and compared the images of adherent macrophages to those acquired with TIR-ROCS. One can see that the image quality concerning resolution and contrast is much lower with RICM, besides the fact that the bright and dark areas are difficult to interpret, since they vary periodically depending on the distance between coverslip and cell surface.

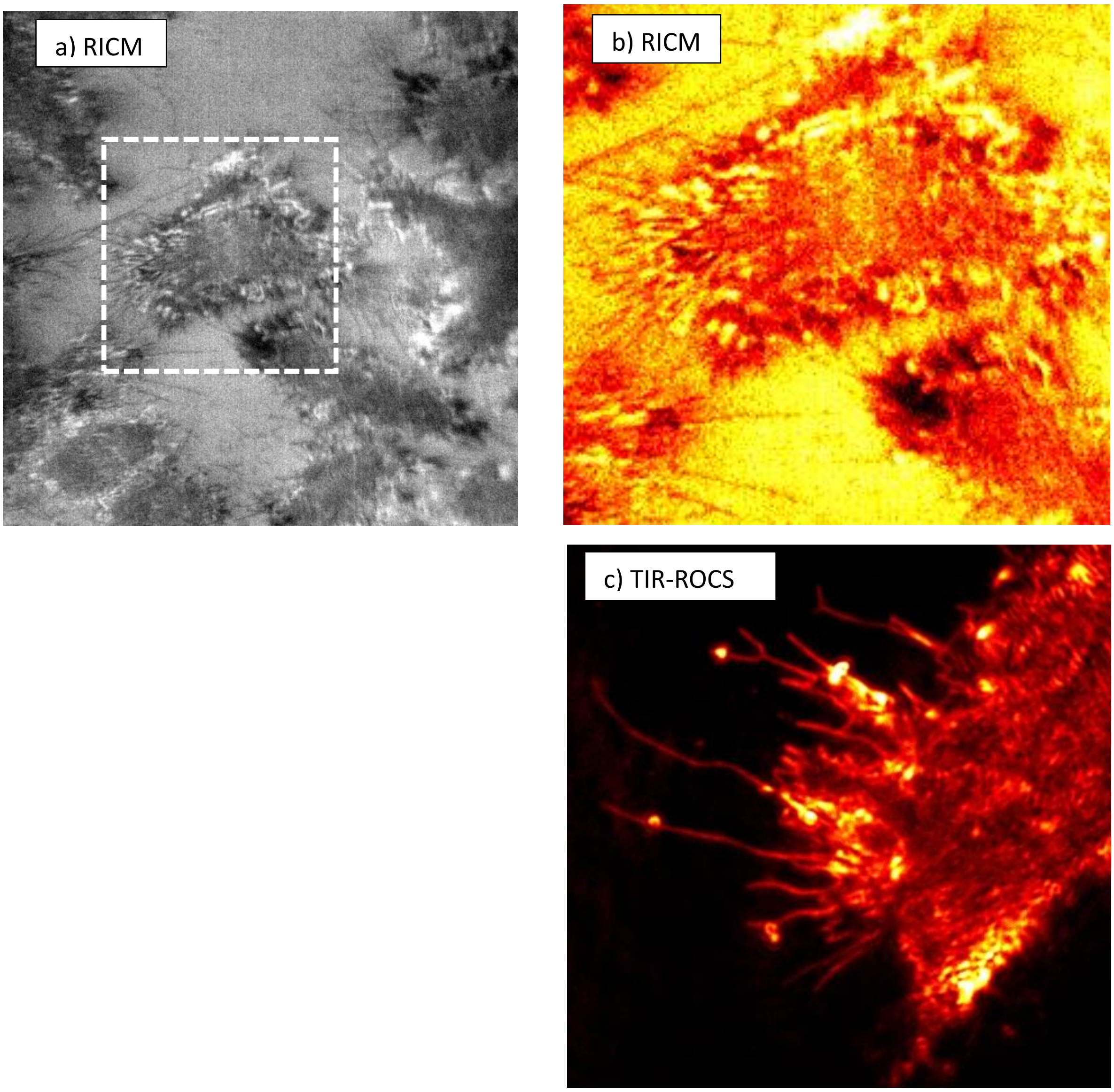

Figure S10 | Comparison of image quality. (a) RICM image of adhering J774 macrophages. **(b)** Colour-coded magnification of the ROI marked in (a). **(c)** TIR-ROCS image.

SUPPORTING REFERENCES

1. Olshausen, P.v. & Rohrbach, A. Coherent Total Internal Reflection Dark Field Microscopy - an approach to label-free imaging beyond the diffraction limit. *Opt. Lett.* **38**, 4066 – 4069 (2013).
2. Kohler, F. & Rohrbach, A. Surfing along Filopodia: A Particle Transport Revealed by Molecular-Scale Fluctuation Analyses. *Biophysical Journal* **108**, 2114-2125 (2015).
3. Lin, C.H. & Forscher, P. Cytoskeletal remodeling during growth cone-target interactions. *The Journal of Cell Biology* **121**, 1369-1383 (1993).
4. Mitchison, T. & Kirschner, M. Cytoskeletal dynamics and nerve growth. *Neuron* **1**, 761-772 (1988).
5. Lehmann, M.J., Sherer, N.M., Marks, C.B., Pypaert, M. & Mothes, W. Actin- and myosin-driven movement of viruses along filopodia precedes their entry into cells. *The Journal of Cell Biology* **170**, 317-325 (2005).
6. Neves, A.A.R. et al. Rotational dynamics of optically trapped nanofibers. *Opt. Express* **18**, 822-830 (2010).
7. Maragò, O.M. et al. Femtonewton Force Sensing with Optically Trapped Nanotubes. *Nano Letters* **8**, 3211-3216 (2008).
